# EXO1 is critical for embryogenesis and the DNA damage response in mice with a hypomorphic *Nbs1* allele

**DOI:** 10.1093/nar/gkv691

**Published:** 2015-07-08

**Authors:** Katrin Rein, Diana A. Yanez, Berta Terré, Lluís Palenzuela, Suvi Aivio, Kaichun Wei, Winfried Edelmann, Jeremy M. Stark, Travis H. Stracker

**Affiliations:** 1 Institute for Research in Biomedicine (IRB Barcelona), Barcelona 08028, Spain; 2 Department of Radiation Biology, Beckman Research Institute of the City of Hope, Duarte, CA 91010, USA; 3 Albert Einstein College of Medicine, Department of Cell Biology, Bronx, NY 10461, USA

## Abstract

The maintenance of genome stability is critical for the suppression of diverse human pathologies that include developmental disorders, premature aging, infertility and predisposition to cancer. The DNA damage response (DDR) orchestrates the appropriate cellular responses following the detection of lesions to prevent genomic instability. The MRE11 complex is a sensor of DNA double strand breaks (DSBs) and plays key roles in multiple aspects of the DDR, including DNA end resection that is critical for signaling and DNA repair. The MRE11 complex has been shown to function both upstream and in concert with the 5′-3′ exonuclease EXO1 in DNA resection, but it remains unclear to what extent EXO1 influences DSB responses independently of the MRE11 complex. Here we examine the genetic relationship of the MRE11 complex and EXO1 during mammalian development and in response to DNA damage. Deletion of *Exo1* in mice expressing a hypomorphic allele of *Nbs1* leads to severe developmental impairment, embryonic death and chromosomal instability. While EXO1 plays a minimal role in normal cells, its loss strongly influences DNA replication, DNA repair, checkpoint signaling and damage sensitivity in NBS1 hypomorphic cells. Collectively, our results establish a key role for EXO1 in modulating the severity of hypomorphic MRE11 complex mutations.

## INTRODUCTION

The maintenance of genomic integrity is critical for development, homeostasis and the suppression of disease (1). DNA lesions are recognized by sensor proteins that activate a DNA damage response (DDR) that includes cell cycle checkpoint activation, modulation of transcription and translation, recruitment or exclusion of DNA repair factors and in some contexts, the activation of apoptosis or senescence programs. DNA double-strand breaks (DSBs) are considered among the most cytotoxic lesions and can give rise to chromosomal rearrangements if not properly metabolized (2). Many genes encoding key players in the DDR to DSBs are mutated in human disorders characterized by genomic instability and DSB sensitivity at the cellular level, and developmental defects in the brain, immune system and germline, as well as increased predisposition to cancer, at the organismal level (1,3). These include the clinically similar Ataxia telangiectasia (AT), AT like disease (ATLD), Nijmegen Breakage Syndrome (NBS) and NBS like disorder (NBSLD), caused by mutations in the *ATM*, *MRE11*, *NBS1* and *RAD50* genes respectively (4–6).

MRE11, RAD50 and NBS1 together form a highly conserved holocomplex, referred to as the MRE11 complex or MRN, that acts as a DSB sensor and plays multiple enzymatic and structural roles in the DDR and DSB repair (4,6,7). MRE11 harbors intrinsic endonuclease and 3′-5′ exonuclease activities involved in DNA end modification and the removal of covalent protein adducts that are regulated by the ATPase activity of RAD50, as well as interactions with NBS1 and CtIP, that may also be an endonuclease (6,8–13). NBS1 promotes the nuclear localization of the MRE11 complex and its accumulation at sites of DNA damage, which requires the N-terminal Forkhead Associated (FHA) and tandem BRCA1 C-terminal (BRCT) domains of NBS1 that function as phosphopeptide recognition modules (4,6,7). The detection of DSBs by the MRE11 complex leads the activation of the Ataxia-telangiectasia mutated (ATM) kinase and Rad3 related (ATR) kinases that together target over 1000 potential substrates (14–18). The MRE11 complex subsequently regulates the ability of ATM to phosphorylate a subset of its targets, including the checkpoint kinase CHK2, that acts in cooperation with ATM to trigger apoptosis (19–22). In response to DSBs, the activation of ATR is temporally distinct from that of ATM and requires nuclease-mediated DNA end resection to generate 3′ ssDNA tails, while in response to other types of replicative lesions, the MRE11 complex can promote ATR activation through the recruitment of its activator TOPBP1 (23–26). ATR is crucial for the activation of the checkpoint kinase CHK1 that influences cell cycle checkpoint responses in S and G2 phase and regulates additional aspects of the DDR, particularly in response to replication stress (27,28).

The MRE11 complex regulates DSB repair pathway choice through its nuclease activities and roles in DNA end resection (29,30). Non-homologous end-joining (NHEJ), which requires ligateable ends, is inhibited by resection, while homologous recombination (HR) requires end resection to generate strand invasion intermediates. The initiation of DNA resection requires the endonuclease activity of MRE11 and is promoted by CtIP (8,11,30–32). Based on extensive genetic analyses and separation of function mutations in yeast, it has been proposed that resection takes place bidirectionally with the 3′-5′ activity of MRE11 resecting towards the end and more extensive 5′-3′ resection, involving primarily the 5′-3′ exonuclease EXO1 or the exo/endonuclease and helicase DNA2, taking place away from the end (31,33–35). Important roles for the BLM and WRN helicases, as well as the ssDNA binding RPA and SOSS1 complexes, in extensive resection have also been described (33,34,36–39). HR is critical for the progression of DNA replication, as well as the restart of stalled replication forks, and both the MRE11 complex and EXO1 localize to the replication fork, suppress replication associated chromosome lesions and influence fork integrity and restart (4,40–51).

Deletion of any MRE11 complex members is embryonic lethal and available data suggests that its role in HR may underlie this requirement (4,6,7,52). EXO1 has also been demonstrated to promote HR and additionally functions in the mismatch repair (MMR) pathway, interacting physically with key MMR pathway components MLH1, MSH2 and MSH3 (50,53,54). However, in contrast to the MRE11 complex components, deletion of EXO1 in mice is viable, although animals exhibit MMR defects, apoptosis defects, sterility due to impaired meiotic recombination and elevated tumor predisposition (53,55,56). The MRE11 complex has been proposed to function both upstream and in tandem with EXO1 during the resection of DSBs (30,33–35), however, deficiency in Mre11 complex members results in strong synthetic phenotypes in Exo1 deficient backgrounds in yeast that are genetically distinct from defects in MMR (57–59). Loss of function studies using siRNA in human cells have generated some conflicting evidence regarding the extent of EXO1's role and dependency on the MRE11 complex for DSB resection and signaling responses. In some cases, depletion of EXO1 has been reported to have mild effects on signaling, repair and sensitivity to damage (60–64), while in others, its depletion has been shown to cause a major effect on DNA repair pathway choice, HR proficiency, sensitivity to IR and camptothecin (CPT) and chromosome integrity (65,66). In addition, the relative roles of the mammalian MRE11 complex and EXO1 in the DSB response and at replication forks remain largely unexplored.

In order to address their genetic and functional relationships, we have examined the interplay between a hypomorphic mutant allele (*Nbs1^ΔB^*) of the MRE11 complex member NBS1 and an *Exo1* null allele during development and in response to diverse DNA lesions at the cellular level. Here, we present evidence that EXO1 deficiency alone causes mild defects in DSB signaling but plays a major role in DSB responses and DNA replication in cells and mice with impaired MRE11 complex function.

## MATERIALS AND METHODS

### Animal generation and husbandry

The generation of *Nbs1^ΔB^* and *Exo1^−^* animals was previously described (55,67). *Nbs1^ΔB^* mice were a kind gift from John H.J. Petrini. Animals were genotyped by PCR, details available upon request. All animals were maintained in strict accordance with the European Community (86/609/EEC) guidelines at the animal facilities in the Barcelona Science Park (PCB). The animal protocols (CEAA13/0008) were approved by the Animal Care and Use Committee of the PCB (IACUC; CEEA-PCB) in accordance with applicable legislation (Law 5/1995/GC; Order 214/1997/GC; Law 1201/2005/SG). All efforts were made to minimize use and suffering.

### Generation and characterization of mouse embryonic fibroblasts (MEFs)

MEFs were isolated from E14.5 embryos as previously described and transformed by transfection with a linearized, origin-less SV40 genome (MEF2 reagent Amaxa Nucleofector; Lonza) (68). Cells were grown routinely in Dulbecco's modified Eagle's medium (DMEM) supplemented with 10%-15% FBS (Hyclone), 2mM Glutamax (Gibco) and 100u/ml penicillin-streptomycin (Gibco). For measurement of growth, MEFs were seeded every three days at the density of 3 × 10^5^ cells per 6-cm plate. The total number of cells was counted at each passage and cumulative cell numbers plotted. For DNA content assessment, cells were resuspended in 300μl PBS containing 25μg/ml propidium iodide (PI) and 0.1mg/ml RNaseA and analysed with FlowJo software following flow cytometry. DNA synthesis rates were assessed by BrdU incorporation and flow cytometry using the BD Pharmingen FITC BrdU Flow Kit (BD Biosciences). The percentage of BrdU positive cells was determined with FlowJo software. The G2/M checkpoint was analyzed by determining the mitotic index before and after treatment using histone H3 phosphorylated on serine 10 (H3S10) as a marker. Briefly, cells were fixed in 70% EtOH, washed and incubated with primary antibody for H3S10 (Millipore), stained with secondary antibody conjugated to FITC and analyzed by flow cytometry. A mitotic ratio (%mitotic cells post mock or IR treatment/%mitotic cells untreated) is presented. For the clonogenic survival assay, transformed MEFs were seeded and treated with the indicated doses of IR, UVC, cisplatin (Ferrer Farma) or CPT (Sigma). 12 days later, colonies were stained with crystal violet and manually counted. Results were normalized for plating efficiencies. For IR and UVC treatments, an X-ray cabinet (Maxishot.200, Krautkramer Forster) or UVC crosslinking oven (Stratalinker, Stratagene) was used.

### Metaphase spread preparations

Cells were seeded at the density of 10^6^ cells per 10-cm plate and the next day colcemid was added to a final concentration of 0.1μg/ml for 30 minutes (transformed MEFs) to 120 min (primary MEFs). MEFs were trypsinized, hypotonically swollen in 0.075 M KCl for 20 min at 37**°**C, fixed (75% MeOH and 25% acetic acid, ice cold) and washed several times in fixative. Metaphase preparations were spread on glass slides, steam treated using an 80**°**C water bath for 3–5 s, heat dried and stained with 5% Giemsa solution (Sigma) and mounted with DPX mounting medium (Panreac). Metaphases were examined and images were taken using a Leica DM6000 microscope with transmitted light illumination. Images were analyzed using Fiji software to quantify the number of metaphases.

### Senescence-associated (SA) β-galactosidase staining

Primary MEFs were seeded at the density of 70 000 cells per 3.5-cm plate. Senescent cells were stained using the Senescence β-Galactosidase Staining Kit (Cell Signalling). Five representative images were taken from diverse areas of the plate, using Nikon TE200 inverted microscope with phase contrast illumination. Images were analyzed using Fiji software to quantify the number of β-galactosidase positive cells.

### Analysis of single strand annealing

The hprtSA-GFP (69) reporter plasmid was linearized by Sac1/Kpn1, and 10μg was electroporated into 1–2.5 × 10^6^ MEFs (0.8 ml total volume, 780 V, 10 μF) per electroporation. Following selection in puromycin (1.5 μg/ml), the puro-resistant clones were pooled and expanded. For the reporter assay, 0.4 × 10^5^ cells were seeded onto 24-well dishes one day prior to transfection with 0.4 μg of the I-SceI expression vector (pCBASce) or the GFP expression vector (pCAGGS-GFP), along with 0.2 μg of empty vector (pCAGGS-BSKX) or Nbs1 expression vector (pCAGGS-Nbs1). All plasmids were previously described (69). Transfections were performed with 1.8 μl Lipofectamine 2000 (Invitrogen) in a total or 0.6 ml volume for 20 h, and subsequently the%GFP+ cells were examined using a CyAN ADP flow cytometer (Dako). Repair frequencies were normalized to transfection by dividing the GFP frequencies from the I-SceI transfections by those of a parallel GFP transfection. Repair frequencies reflect the mean of at least six independent transfections, and the error bars are the SD. We also normalized the repair frequencies for the *Nbs1^ΔB/ΔB^ Exo1^−/−^* cells percentage of cells in the S/G2 phase population, as determined by BrdU and PI staining and flow cytometry. To examine NBS1 expression, cells were extracted 2 days after transfection using NETN (20mM Tris, pH 8, 100 mM NaCl, 1 mM EDTA, 0.5% IGEPAL, 1.25 mM DTT, and Roche Protease Inhibitor Mixture) using several freeze/thaw cycles. Blots of these extracts were probed with antibodies against buffer and several freeze thaw cycles, and were probed with Nbs1 (Bethyl A301-284A) and Actin (Sigma A2066) antibodies.

### DNA fiber assay

2 × 10^5^ MEFs were seeded per 6-cm plate and pulse-labeled with 25 μM 5-chloro-2′-deoxyuridine (CldU, Sigma–Aldrich) followed by 250 μM 5-iodo-2′-deoxyuridine (IdU, Sigma–Aldrich) with the durations indicated in the figure legends. For replication restart, cells were treated with 1 mM hydroxyurea (HU, Sigma–Aldrich) for 15 min after 20 min CldU labelling, followed by 40 min IdU labeling. Cells were harvested and resuspended in ice-cold PBS at 10^6^ cells/ml. 2 μl of cell suspension was dropped on the top of the microscope slide (Superfrost, Fisher Scientific), dryed for 6 min, 7 μl of spreading buffer (200 mM Tris–HCl pH 7.4, 50 mM EDTA, 0.5% SDS) was added to each drop, mixed by stirring with the pipette tip, followed by 2 min of incubation. Chromatin was spread by tilting the slides (20–30^o^) and they were air-dryed and fixed in acetic acid:methanol (1:3) for 10 min. For DNA denaturation, slides were washed 2× in H_2_O for 5 min, 1× with 2.5 M HCl, denatured with 2.5M HCl for 1 h, and rinsed two times with PBS. For immunodetection, slides were blocked for 1 h in blocking solution (PBS, 1% BSA, 0.1% Tween20) and incubated overnight at 4°C with a mix of rat anti-BrdU monoclonal antibody (1:500 in blocking solution, AbD Serotec) that recognizes CldU, and mouse anti-BrdU monoclonal antibody (1:500 in blocking solution, Becton Dickinson) that recognizes IdU. Slides were washed three times with PBS and fixed with 4% formaldehyde for 10 min, following by three times washing with PBS and three times with blocking solution. The slides were then incubated at 37°C for 2 h with a mix of an AlexaFluor 555-conjugated goat anti-rat IgG (1:500 in blocking solution, Life Techologies) and an AlexaFluor 488-conjugated goat anti-mouse IgG (1:500 in blocking solution, Life Techologies). Slides were washed five times with PBS, dried, and mounted with Vectashield mounting medium (Vector Laboratories). Fibers were examined with Leica TCS SPE confocal microscope using 63× objective. The lengths of CldU-labeled (red) and IdU-labeled (green) patches were measured using Fiji software, and micrometer values were converted into kilobases using the conversion factor 1 mm = 2.59 kb (70).

### Total protein isolation and western blotting

Cells were lysed with TNG-150 lysis buffer containing 50 mM Tris–HCl, 150mM NaCl, 1% Tween 20, 0.5% NP-40, 1× Phosphatase Inhibitor Cocktail 2 (Sigma–Aldrich) and 1× Protease Inhibitor Cocktail (Roche). Protein concentration was quantified with DC Protein Assay Kit (Bio-Rad). 30–80 μg of total protein was separated by SDS-PAGE (8–12% gels) or NuPAGE Novex Tris-Acetate SDS (3–8% gels, Life Technologies) followed by transfer to Immobilon-P PVDF membrane (Merck-Millipore). For blocking solution, 5% milk in PBST was used, membranes were incubated in primary antibody at 4°C overnight in secondary antibody 1 h at room temperature. ECL reagent (Amersham) with X-ray film (Fujifilm) were used to detect the signal.

**Table:** 

Antibody	Source	Dilution
ATM	Sigma–Aldrich A1106	1:3000
P-ATM (Ser1981)	Cell Signalling 4526	1:3000
CHK1	Santa Cruz sc7898	1:500
P-CHK1 (Ser345)	Cell Signalling 2341	1:1000
CHK2	Merck–Millipore 05–649	1:500
RPA	Merck–Millipore NA19L	1:1000
P-RPA (S4/S8)	Bethyl A300–245A	1:2000
CtIP	Santa Cruz sc271339	1:500
NBS1	Novus NB100–143	1:1000
EXO1	Bethyl A302–640A	1:5000
α-Tubulin	Sigma–Aldrich T5168	1:8000

### siRNA-mediated knockdown of proteins

Small interfering (si)RNAs were designed using the siRNA Selection Program (Whitehead Institute for Biomedical Research) and manufactured by Invitrogen. MEFs were transfected using Lipofectamine RNAiMAX (Invitrogen) 2× within a 24-h interval. All experiments were done 48 h after the first transfection. siRNA to GFP was used as a control.

**Table:** 

Gene	RNA sequence
*GFP*	GGC UAC GUC CAG GAG CGC CGC ACC [dT][dT]
*Mus81-siRNA1*	GUG AAG CGA ACC AUG GAU A [dT][dT]
*Mus81-siRNA2*	CUC UUG AGC ACC AUC AAG U [dT][dT]

### Immunofluorescence

Cells were seeded on 8-well dish with the density of 15 000 cells/well. Cells with fixed in 4% formaldehyde for 10 min in room temperature, followed by permeabilization with PBS + 0.2% Triton X-100 for 10 min at room temperature. Cells were incubated with DAPI for 1 h, mounted in Vectashield mounting media and analyzed with Leica DM6000 microscope.

### Kinase inhibitor and phosphatase treatment

Cells were pretreated with DNA-PKcs (NU7441, Tocris) at 12 μM and ATM inhibitors (KU55933, Sigma) at 10 μM for 30 min prior to treatment. λ-phosphatase (NEB) was added at 1 μl per 25 μl lysate (8 units/ μg of protein) and incubated at 30°C for 30 min.

## RESULTS

### Deletion of *Exo1* leads to embryonic lethality in hypomorphic *Nbs1* mice

While the deletion of *Nbs1* leads to early embryonic lethality, mice expressing a hypomorphic allele of *Nbs1*, *Nbs1^ΔB^*, are viable, show normal longevity and are not predisposed to malignancy (67). The *Nbs1^ΔB^* mutation leads to the production of a truncated NBS1 protein, termed p80, that is expressed at lower levels and lacks the FHA and first BRCT domain in the N-terminus, but maintains interactions with the MRE11 and RAD50 proteins (4). At the cellular level, the *Nbs1^ΔB^* allele impairs DSB checkpoint signaling and confers hypersensitivity to a variety of DSB inducing agents (19,20,67,71,72). However, unperturbed *Nbs1^ΔB/ΔB^* mice and cells show minimal spontaneous DNA damage or cell death compared to *Mre11* or *ATM* mutants, and mice are not prone to malignancy, making them a useful tool for probing genetic interactions with the MRE11 complex-ATM signaling axis (19,20,72,73). We therefore chose the *Nbs1^ΔB^* allele to examine the genetic and functional relationship of the MRE11 complex with EXO1.

**Figure 1.**
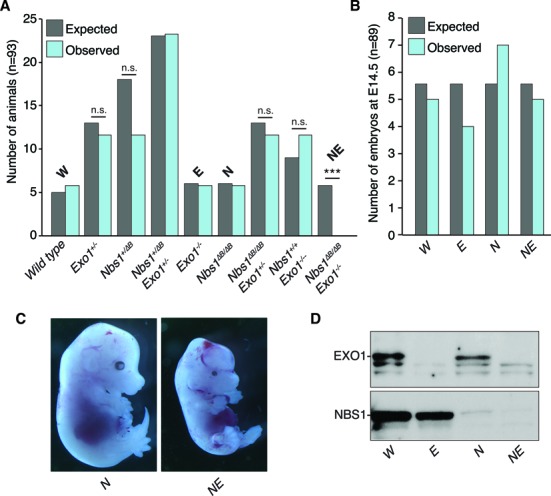
Deletion of Exo1 leads to embryonic lethality in hypomorphic *Nbs1* mice. (**A**) Graph of expected and observed live born pups from double heterozygous breedings based on normal Mendelian inheritance (*n* = 93). For brevity, the primary genotypes are abbreviated as follows: wild type = *W*, *Nbs1^ΔB/ΔB^* = *N*, *Exo1^−/−^* = *E*, *Nbs1^ΔB/ΔB^Exo^−/−^* = *NE*. Statistical analysis was performed using an unpaired *t*-test. ***P < 0.0001 and n.s. = not significant. (**B**) Graph of expected and observed E14.5 embryos from double heterozygous breedings based on normal Mendelian inheritance (*n* = 89). (**C**) Representative images of E14.5 embryos of the indicated genotype. (**D**) Western blotting of NBS1 and EXO1 from embryonic fibroblast cultures derived from E14.5 embryos.

To determine if the loss of EXO1 modified the phenotypes of *Nbs1^ΔB^*, we crossed *Nbs1^+/ΔB^ Exo1^+/−^* double heterozygous animals to generate double mutants, as homozygosity of either allele leads to subfertility or infertility respectively (53,67). Despite observing the expected distribution of both single homozygous alleles, we observed no double mutant pups, suggesting embryonic lethality (Figure 1A).

The examination of E14.5 embryos revealed that double mutant embryos appeared with the expected frequency (Figure 1B) but were severely runted and showed developmental defects, including runting and malformation of the cranium (Figure 1C). Western blotting confirmed the expected protein status of both NBS1 and EXO1 in cell cultures from the double mutant embryos (Figure 1D). These data indicated that EXO1 is essential for mammalian development when non-essential functions of the MRE11 complex functions are compromised.

### EXO1 suppresses chromosomal instability in *Nbs1* mutants

**Figure 2.**
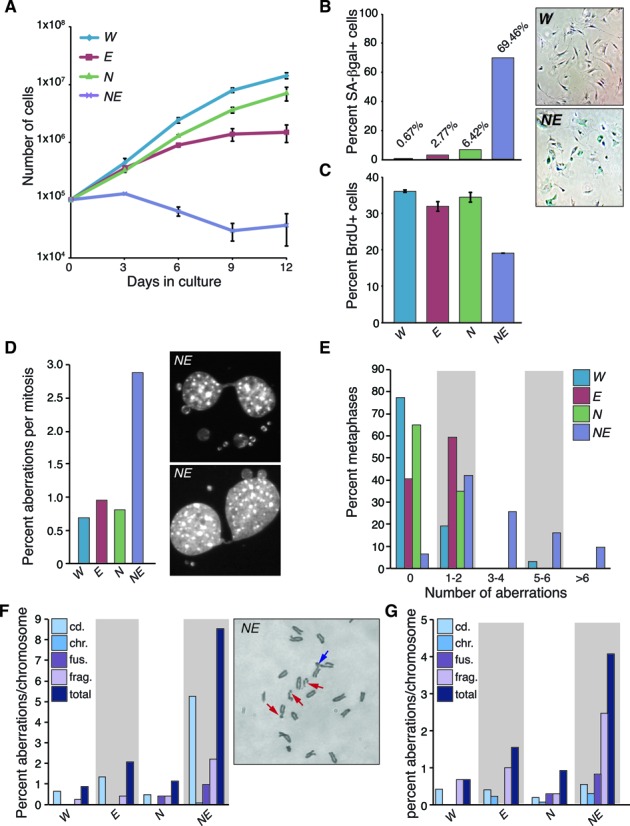
EXO1 suppresses chromosomal instability in *Nbs1* mutants. (**A**) Cell growth determined using a modified 3T3 protocol. Primary MEFs were counted and replated every 3 days and cumulative growth was plotted for the indicated genotypes. The genotypes are abbreviated throughout the figure as follows: wild type = *W*, *Nbs1^ΔB/ΔB^* = *N*, *Exo1^−/−^* = *E*, *Nbs1^ΔB/ΔB^Exo^−/−^* = *NE*. (**B**) Premature senescence in *NE* double mutant cultures. Passage 6 MEFs were stained for senescence associated (SA) β-galactosidase activity and counted under magnification. The percentage of SA β-gal positive cells is plotted. (**C**) DNA synthesis is reduced in *NE* cell cultures. Passage 2 MEFs were pulsed with BrdU for 4 h and the percentage of BrdU positive cells was measured by flow cytometry combined with propidium iodide (PI) staining. (**D**) Increased anaphase bridges and micronuclei are evident in *NE* cultures. Quantification of the percentage of aberrant mitoses in primary cell cultures. A minimum of 100 mitoses are scored in each case. (**E**) Increased chromosomal instability in primary, early passage (p2-p3) *NE* cell cultures. The number of metaphase aberrations per metaphase were scored and binned as indicated in the figure. A higher percentage of *NE* metaphases show more than two aberrations per metaphase. (**F**) Plot of chromosomal aberration types (cd.=chromatid break, chr.=chromosome break, fus.=fusion, frag.=fragment) scored from multiple primary cell cultures depicted as percent aberrations per chromosome. *NE* cultures have particularly high numbers of chromatid breaks. Examples of aberrations scored in a partial *NE* metaphase are shown in the right panel. Red arrows indicate cd aberrations and blue arrow indicates a fusion. Additional examples are provided in Supplementary Figure S1. (**G**) Plot of chromosomal aberration types (as in (F)) identified in transformed cell cultures of the indicated genotypes. Total aberrations remain higher in *NE* cultures although chromosome fragments were the predominant lesion identified.

In order to better understand the genetic interaction between *Exo1* deficiency and the *Nbs1^ΔB^* allele, we generated primary mouse embryonic fibroblasts (MEFs) from E14.5 embryos. *Nbs1^ΔB/ΔB^ Exo1^−/−^* cultures showed a strong growth defect compared to wild type or single mutants and displayed morphological changes indicative of premature senescence (Figure 2A and B). Consistent with this, we found that DNA synthesis was strongly reduced in early passage *Nbs1^ΔB/ΔB^ Exo1^−/−^* cell cultures (Figure 2C) and by passage 6, nearly 70% of the double mutant cells were senescent, compared to <7% in *Nbs1^ΔB/ΔB^* (Figure 2B). To determine if increased genomic instability could explain the premature senescence phenotype of the *Nbs1^ΔB/ΔB^ Exo1^−/−^* cultures, we examined mitotic figures by DAPI staining and scored the frequency of anaphase bridges and micronuclei. While a basal level of both aberrations was observed in all genotypes, *Nbs1^ΔB/ΔB^ Exo1^−/−^* cultures showed a nearly 3-fold increase compared to the other backgrounds (Figure 2D). As the high frequency of mitotic aberrations suggested that cells were entering mitosis with chromosomal damage, we next examined chromosomal aberrations in early passage metaphase preparations. While only a small increase in aberrations were identifiable in the single mutants, *Nbs1^ΔB/ΔB^ Exo1^−/−^* cells displayed a high level of metaphase aberrations in the absence of exogenous damage, with a high percentage of metaphases harboring multiple aberrant figures (Figure 2E). In particular, chromatid breaks, indicative of replication associated DNA damage, were the most abundant lesion observed (Figure 2F and Supplementary Figure S1). The impaired proliferative capacity and high levels of chromosomal instability in the double mutant cells indicated that EXO1 suppressed replication associated damage and/or promoted efficient repair or checkpoint responses in the hypomorphic *Nbs1^ΔB/ΔB^* background. As the strong growth defect of *Nbs1^ΔB/ΔB^ Exo1^−/−^* cells made further expansion for functional analysis difficult, we transformed single and double mutant cells by the transfection of an origin-less SV40 genome (68). Transformed *Nbs1^ΔB/ΔB^ Exo1^−/−^* cells continued to exhibit increased levels of spontaneous chromosomal aberrations, albeit to a lesser extent than in primary cell cultures, and grew more slowly than single mutants (Figure 2G and Supplementary Figure S2).

### EXO1 is required for ATM/ATR-dependent checkpoint signaling in NBS1 mutant cells

**Figure 3.**
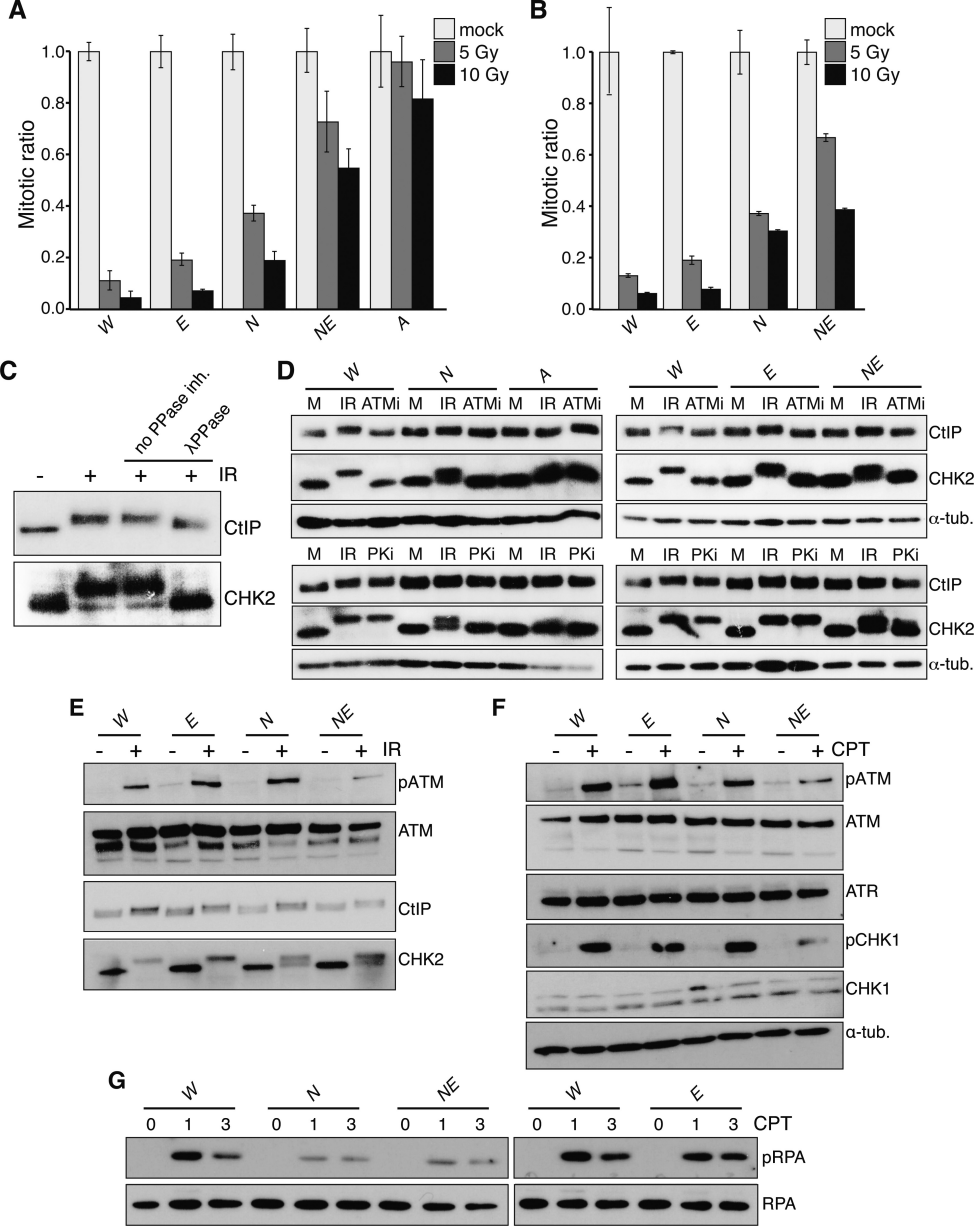
EXO1 is required for ATM/ATR signaling and the G2/M checkpoint in NBS1 mutant cells. (**A**) G2/M checkpoint activation in primary MEFs (p2-p3) 1 h after the indicated doses of IR scored by flow cytometric determination of the percentage of phospho-H3S10 positive cells with a G2 DNA content (determined by PI staining). The mitotic ratio is plotted (mock or IR treated cells, normalized to mock treated). The genotypes are abbreviated throughout the figure as follows: wild type = *W*, *Nbs1^ΔB/ΔB^* = *N*, *Exo1^−/−^* = *E*, *Nbs1^ΔB/ΔB^Exo^−/−^* = *NE* and *Atm^−/−^* = *A*. (**B**) Checkpoint activation in transformed MEFs 1 hour after the indicated doses of IR, plotted as in (A). (**C**) The mobility shift of CHK2 and CtIP induced by IR is phosphorylation dependent. Lysates containing protease and phosphatase inhibitors were mock or IR treated and a mobility shift is evident for both proteins. Lysates from IR treated cells were harvested without phosphatase inhibitors (second 2 lanes) and treated with λ-phosphatase (last lane). (**D**) Hyperphosphorylation of CHK2 and CtIP is ATM dependent. Cell cultures of the indicated genotypes were pre-treated for 30 min with inhibitors of ATM (Atmi) or DNA-PKcs (PKi), mock or IR treated (10 Gy) in the presence of inhibitors and harvested 1 h post IR. The increased mobility of CHK2 and CtIP is lost in *Atm^−/−^* deficient cells or following the addition of ATMi. (**E**) Western blotting of the indicated proteins 1 h after 10 Gy IR treatment. (**F**) Western blotting of the indicated proteins 1 h after 1 mM CPT treatment. (**G**) Western blotting of the indicated proteins 1 and 3 h after 1 mM CPT treatment.

In response to IR, the MRE11 complex is required for efficient G2/M checkpoint initiation through the activation of the ATM kinase (17,18) and subsequent activation of ATR and CHK1 kinases, that are influenced by the activities of both the MRE11 complex and EXO1 (23,34,74). To determine if the loss of EXO1 itself affected the G2/M checkpoint response, or modified the defect in *Nbs1* mutants, we examined checkpoint induction following IR treatment. As previously reported, we observed a mild defect in *Nbs1^ΔB/ΔB^* mutants compared to the severe defect of *Atm^−/−^* cells (71), while EXO1 loss alone did not detectably affect checkpoint induction (Figure 3A). However, we observed that the checkpoint defect of *Nbs1^ΔB/ΔB^* mutants was strongly enhanced by the loss of EXO1 in both primary and transformed *Nbs1^ΔB/ΔB^ Exo1^−/−^* cell cultures (Figure 3A and B). This indicated that while EXO1 status had no detectable influence on checkpoint activation in the presence of functional MRE11 complex, it was required for much of the checkpoint response observed in hypomorphic *Nbs1^ΔB/ΔB^* cell cultures.

To understand more clearly how EXO1 loss influenced the checkpoint in *Nbs1^ΔB/ΔB^*, we examined the signaling events elicited by IR, 1 h following IR exposure when checkpoint activity is maximal. IR rapidly induces ATM autophosphorylation, which can be detected with phospho-specific antibodies against S1987, as well as the increased mobility of both CHK2 and CtIP. As reported previously, increased mobility of both the CHK2 and CtIP proteins was dependent on phosphorylation, as the addition of λ-phosphatase to extracts reduced their mobility to that of untreated cells, and their increased mobility was abolished in cells lacking ATM or treated with an ATM inhibitor (Figure 3C and D) (75,76). In cells expressing the *Nbs1^ΔB/ΔB^* allele, CHK2 phosphorylation was also sensitive to treatment with a DNA-PKcs inhibitor, indicating that DNA-PKcs may promote CHK2 phosphorylation in an ATM dependent manner in this background or that this inhibitor has some off-target effects (Figure 3D).

We found that neither the loss of EXO1 nor the *Nbs1^ΔB^* mutation alone affected ATM activation, as evidenced by levels of S1987 phosphorylation similar to that of wild type cells (Figure 3E). However, in *Nbs1^ΔB/ΔB^ Exo1^−/−^* cultures, ATM activation was markedly reduced (Figure 3E). We therefore examined the hyperphosphorylation of the ATM targets CHK2 and CtIP, both of which have been characterized as dependent on NBS1 as a mediator for their phosphorylation (21,22,76). The hyperphosphorylation of CHK2 was unaffected by EXO1 loss and impaired to a similar extent in both *Nbs1^ΔB/ΔB^* and *Nbs1^ΔB/ΔB^ Exo1^−/−^* cell cultures (Figure 3E, bottom panel and Supplementary Figure S3).

Surprisingly, CtIP hyperphosphorylation occurred similarly in any of the single or compound mutations tested, indicating that this could occur in the absence of the NBS1 FHA and BRCT domains, that are deleted in the *Nbs1^ΔB^* allele, and full activation of ATM, which is consistent with the proposed role for cyclin-dependent kinases (CDKs) in priming CtIP hyperphosphorylation (Figure 3E) (76). These results suggest that in contrast to CHK2, CtIP hyperphosphorylation by ATM is unaffected by the *Nbs1^ΔB^* mutation or EXO1 status. In either case however, the absence of EXO1 did not influence their ATM-dependent phosphorylation, regardless of NBS1 status.

As both the MRE11 complex and EXO1 have been implicated in DNA replication and the control of DNA resection, we next examined signaling following exposure to CPT, a topoisomerase I inhibitor that generates DNA damage primarily during S-phase and results in more extensive resection than IR (62,77). CPT treatment led to rapid ATM and ATR activation, the latter assayed indirectly by the S345 phosphorylation of its target, CHK1 (26). Comparable levels of ATM and CHK1 activation were observed in wild type and *Nbs1^ΔB/ΔB^* or *Exo1^−/−^* single mutant cultures while both phosphorylation events were reduced in *Nbs1^ΔB/ΔB^ Exo1^−/−^* cultures (Figure 3F). In contrast, the phosphorylation of RPA was primarily dependent on NBS1 status as it was reduced to a similar extent in *Nbs1^ΔB/ΔB^* and *Nbs1^ΔB/ΔB^ Exo1^−/−^* cells and only mildly decreased in *Exo1^−/−^* cultures (Figure 3G). These data indicated that in response to both IR and CPT, EXO1 was essential for efficient ATM activation when MRE11 complex functions were compromised. However, EXO1 loss does not further impair the phosphorylation of some targets that require the MRE11 complex as a mediator, such as CHK2 or RPA, the latter proposed to be a marker of global resection levels (11,62).

### EXO1 influences DNA repair and DNA replication in *Nbs1* mutants

**Figure 4.**
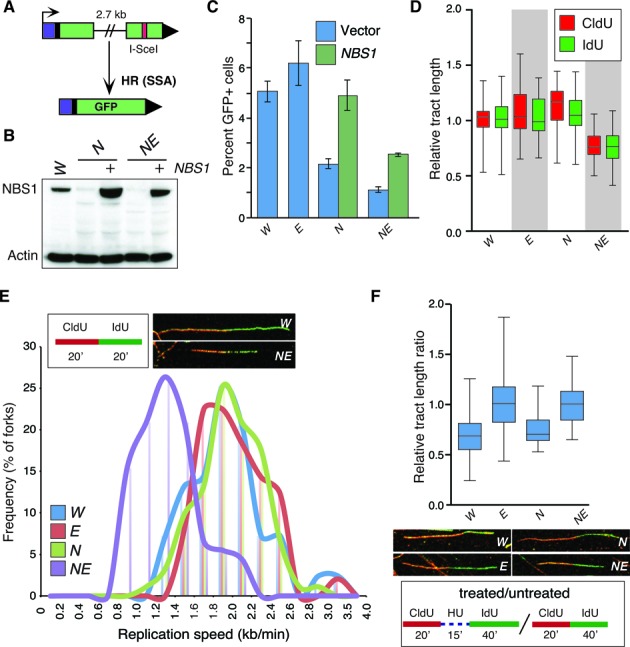
EXO1 influences DNA repair and DNA replication in *Nbs1* mutants. (**A**) Schematic illustration of the SA-GFP based SSA assay. (**B)** Western blotting of cells transfected with or without a vector expressing human NBS1. The genotypes are abbreviated throughout the figure as follows: wild type = *W*, *Nbs1^ΔB/ΔB^* = *N*, *Exo1^−/−^* = *E*, and *Nbs1^ΔB/ΔB^ Exo^−/−^* = *NE*. (**C**) Quantification of SSA mediated repair plotted as the percentage of GFP positive cells. Values for NE are corrected for the reduced percentage of cells in S/G2 determined by BrdU and PI staining (mean 58% compared to a mean of 73% in the other genotypes). The fold rescue with *NBS1* expression is the same in *N* and *NE* (2.2 fold). (**D**) Measurement of replication tract lengths following CldU or IdU pulse labeling. (**E**) Calculation of replication fork velocity in the indicated genotypes (as described in ‘Materials and Methods’ section). Examples of representative forks from *W* and *NE* cultures are shown for comparison. (**F**) Assessment of replication fork restart following 1 mM HU treatment using the indicated scheme. Relative tract ratio is calculated by dividing the length of the IdU tract by that of the CldU tract ( = 2 under unperturbed conditions). Thus, higher values indicate faster restart following HU removal.

The analysis of signaling defects in *Nbs1^ΔB/ΔB^ Exo1^−/−^* cultures suggested that ATM and ATR pathways were further impaired in *Nbs1^ΔB/ΔB^* cells by the loss of EXO1 but did not provide a clear indication as to whether this was due to an effect on DNA resection. To address this further, we examined resection dependent DNA repair using an integrated reporter for single strand annealing (SSA), a sub-pathway of homologous recombination that requires extensive resection. For this, we used the SA-GFP reporter that contains two homologous fragments of a GFP expression cassette separated by a marker gene (puromycin), with a recognition site for the I-SceI endonuclease within the 3′ fragment (Figure 4A). SSA repair of the I-SceI-induced DSB that uses 0.28 kb homologous repeats in the GFP fragments to bridge the DSB restores GFP expression and causes a 2.7 kb deletion between the repeats (69). Accordingly, this event requires at least 2.7 kb of end resection to reveal the homology used during repair.

We integrated this reporter into single and double mutant MEFs, expressed I-SceI and determined the frequency of GFP positive cells, normalized to parallel transfections with a GFP expression vector. For the *Nbs1^ΔB/ΔB^* cells, we also performed a parallel co-transfection of I-SceI with an *NBS1* expression vector (Figure 4B). Since we found that the *Nbs1^ΔB/ΔB^ Exo1^−/−^* cells showed a reduced percentage of cells in S/G2 (mean 58% compared to a mean of 71–73% in the other genotypes), we normalized the SSA values to reflect this change in cell cycle profile (Supplementary Figure S4). In these experiments, no reduction of SSA was observed in *Exo1^−/−^* cultures compared to wild type controls (Figure 4C). However, *Nbs1^ΔB/ΔB^* and *Nbs1^ΔB/ΔB^ Exo1^−/−^* cultures showed a significant reduction in SSA, compared to wild type (2.3-fold and 4.5-fold, respectively, p<0.0001). Notably, SSA was lower in *Nbs1^ΔB/ΔB^ Exo1^−/−^* than *Nbs1^ΔB/ΔB^* cells (1.9-fold, *P* < 0.0001), although transient *Nbs1* expression led to a similar increase in SSA in both genotypes (2.2-fold) (Figure 4C). These findings indicate that, in contrast to what has been observed in yeast, EXO1 is not required for SSA in NBS1 proficient mammalian cells (33). However, in *Nbs1^ΔB/ΔB^* mutants, EXO1 appears to function in promoting an HR event that requires extensive resection. Although we cannot strictly rule out that reduced growth and increased spontaneous DNA damage of *Nbs1^ΔB/ΔB^ Exo1^−/−^* cells also plays an indirect role in suppressing efficient SSA.

Homologous recombination is an important mechanism for the restart or bypass of stalled replication forks (78). To determine if the defect we observed in SSA assays was potentially indicative of problems with other end-resection dependent processes that may impact replication fork progression or fork restart, we first examined replication fork progression using dual labeling with the BrdU analogs IdU and CldU and chromatin fiber spreads. The measurement of replication fork tract length was unaffected by the analog used and the mean tract length was similar in wild type or either single mutant (Figure 4D). However, mean replication tract length and replication fork speed showed a pronounced reduction in *Nbs1^ΔB/ΔB^ Exo1^−/−^* cultures (Figure 4D and E). This is consistent with EXO1 being largely dispensable for normal replication fork progression but playing a crucial role in maintaining fork speed when MRE11 complex functions are compromised, potentially through the modulation of resection-dependent repair pathways and/or the resolution of aberrant replication intermediates.

We also considered that this could represent a role for EXO1 in replication fork restart that was similar or redundant to that described for the MRE11 complex (79). To address this, we examined the ability of stalled replication forks to restart in the single and double mutant cultures by first pulsing with CldU and then arresting forks with hydroxyurea (HU) for 15 min. We then washed out the HU and pulsed with Idu for 40 min and compared the replication tract length ratio to assess the relative speed of fork restart. We found that while fork restart was comparable to wild type in *Nbs1^ΔB/ΔB^* cultures, the loss of EXO1 in either background led to a more rapid restart of forks, evidenced by a higher average tract length ratio (Figure 4F). This suggested that despite MRE11 complex status, EXO1 normally restrains the restart of stalled forks, independently of its influence on DSB resection or the overall speed of replication forks prior to stalling.

### Differential effects of EXO1 loss on the damage sensitivity of *Nbs1* mutants

**Figure 5.**
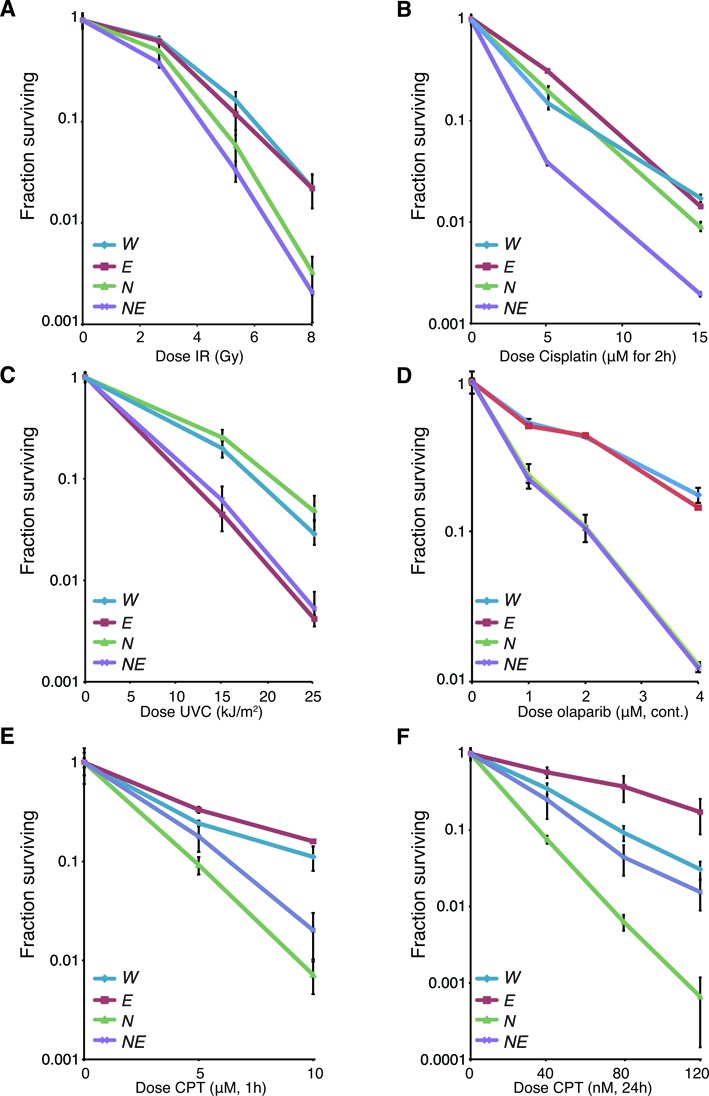
Differential effects of EXO1 loss on the damage sensitivity of *Nbs1* mutants. Sensitivity of the cell cultures of the indicated genotypes to DNA damaging agents or PARP inhibitor (Olaparib) using the clonogenic survival assay. The genotypes are abbreviated in each case as follows: wild type = *W*, *Nbs1^ΔB/ΔB^* = *N*, *Exo1^−/−^* = *E*, and *Nbs1^ΔB/ΔB^ Exo^−/−^* = *NE*. (**A**) IR sensitivity following treatment with 2, 5 or 8 Gy. (**B**) Cisplatin sensitivity following treatment at the indicated doses for 2 h. (**C**) UVC sensitivity after treatment with 15 or 25 J/m^2^. (**D**) Olaparib sensitivity following continuous treatment with the indicated doses. (**E**) CPT sensitivity (high) following treatment with the indicated doses for 1 h. (**F**) CPT sensitivity (low) following treatment with the indicated doses for 24 hours.

As EXO1 loss exacerbated the G2/M checkpoint and SSA defects of *Nbs1^ΔB/ΔB^* cultures and led to impaired DNA replication fork progression, we considered that EXO1 status may strongly influence the level and spectrum of DNA damage sensitivity in *Nbs1^ΔB/ΔB^* cells. To address this, we examined the sensitivity of MEF cultures of each genotype to IR, UVC, cisplatin, the poly-ADP ribose polymerase (PARP) inhibitor Olaparib and both low and high doses of CPT that generate different spectrums of DNA lesions, the latter treatment inducing much higher levels of DSBs (Figure 5A-F and Supplementary Figure S5) (80). In response to IR, *Nbs1^ΔB/ΔB^ Exo1^−/−^* cells were more sensitive than *Nbs1^ΔB/ΔB^* and this was even more apparent following cisplatin treatment (Figure 5A and B). In contrast, sensitivity to UVC was influenced only by EXO1 status, while sensitivity to the PARP inhibitor Olaparib, that is considered a diagnostic for homologous recombination deficiency, was only dependent on the *Nbs1^ΔB^* allele (Figure 5C and D) (81–84). *Nbs1^ΔB/ΔB^* mutants were highly sensitive to both low and high doses of CPT but surprisingly, we found that, particularly with low dose CPT treatment, EXO1 loss notably improved the survival of both wild type and *Nbs1^ΔB/ΔB^* cultures (Figure 5E and F). These results suggested that while EXO1 enhanced the survival of *Nbs1^ΔB/ΔB^* cells after IR or cisplatin treatments, it promoted toxicity in *Nbs1^ΔB/ΔB^* and to a lesser extent, wild type cells, following CPT treatment.

### EXO1 promotes sensitivity to low dose camptothecin

**Figure 6.**
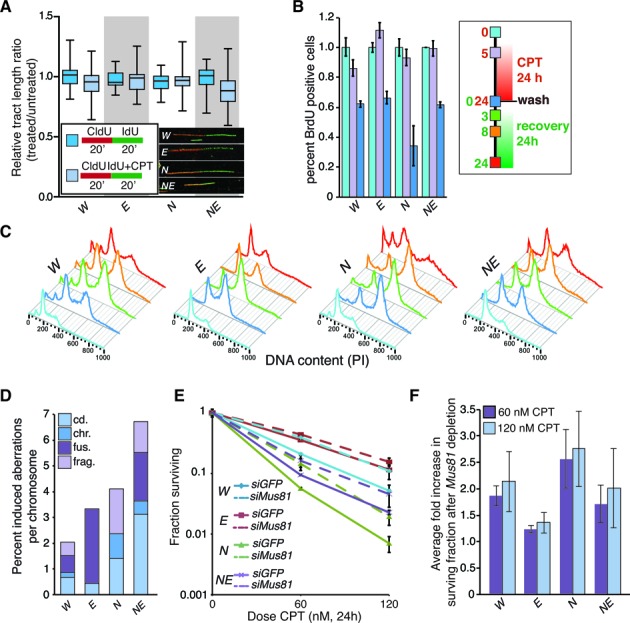
EXO1 modifies the cellular response to low dose camptothecin. (**A**) Measurement of replication fork tracts upon CPT treatment in the indicated genotypes. The tract length ratios (IdU/CldU) were not significantly different with or without 80 nM CPT treatment in the initial 20 min. The genotypes are abbreviated in each case as follows: wild type = *W*, *Nbs1^ΔB/ΔB^* = *N*, *Exo1^−/−^* = *E*, and *Nbs1^ΔB/ΔB^ Exo^−/−^* = *NE*. (**B**) Analysis of BrdU incorporation at the indicated times post CPT addition by flow cytometry. The percentage of cells incorporating BrdU drops more dramatically in *Nbs1^ΔB/ΔB^* cultures after 24 hours of treatment compared to other genotypes. Time points (0, 5 and 24 h post CPT treatment) were assessed (legend corresponds to panels B and C). (**C**) Cell cycle profiles of cell cultures of the indicated genotypes following CPT treatment and withdrawal. (**D**) Metaphase aberrations induced by 50 nM CPT treatment and 4 h of recovery in the indicated genotypes. (**E**) Sensitivity of cell cultures of the indicated genotypes transfected with siRNA (siGFP as a control or 2 siRNAs against *Mus81*) to the indicated dose of camptothecin using the colony formation assay. Average results from triplicate experiments with 2 different *Mus81* siRNAs (*n* = 6) are plotted and standard deviation indicated. (**F**) Average fold increase in survival following the depletion of MUS81 calculated by dividing the fraction surviving values of the MUS81 siRNA treated cells by the siGFP controls for each of the genotypes.

We next more carefully examined the effects of low dose CPT on DNA replication in the different genetic backgrounds. The length of CldU and IdU labeled tracts was similar to untreated cells immediately following low dose (80 nM) CPT treatment, indicating that there were not global changes in replication fork speed or detectable fork stalling upon CPT treatment in any of the genotypes at early times (Figure 6A). Attempts to measure replication tracts at later time points failed due to aggregation of chromatin fibers, potentially a result of covalent protein attachments or torsional issues (Supplementary Figure S6). Instead we interrogated DNA synthesis using BrdU pulse labeling 5 and 24 h following CPT addition (Figure 6B). Similar to the fork progression results immediately following CPT addition (Figure 6A), we did not observe an appreciable reduction in BrdU incorporation 5 hours following CPT addition indicating that there was not a global change in the number of cells entering S-phase and incorporating BrdU. However, by 24 h after CPT addition, BrdU incorporation was reduced in all genotypes. The highly sensitive *Nbs1^ΔB/ΔB^* cultures were the most severely affected, showing nearly an 80% reduction in DNA synthesis compared to around 40% in the other genotypes (Figure 6B). The analysis of cell cycle progression by PI staining at various times following low dose CPT treatment showed that DNA fragmentation (sub G1) was increased in the *Nbs1^ΔB/ΔB^* cultures and reduced in *Nbs1^ΔB/ΔB^ Exo1^−/−^* to levels comparable to that of wild type or *Exo1^−/−^* (Figure 6C). This did not directly correlate with the levels of metaphase aberrations, as we found that there were higher numbers of chromosomal aberrations induced by CPT in *Nbs1^ΔB/ΔB^ Exo1^−/−^* cultures relative to the other genotypes (Figure 6D). Compared to wild type, a mild increase in aberrations was observed in *Exo1^−/−^* cultures and a stronger increase was seen in *Nbs1^ΔB/ΔB^* cultures but notably, chromosome fusions, that are likely to be a result of non-homologous end-joining mediated repair, were not observed in the *Nbs1^ΔB/ΔB^* mutants and elevated in cells lacking EXO1 (Figure 6D).

Structural studies using electron microscopy (EM) have demonstrated that low dose CPT treatment produces fewer DNA breaks than acute treatment with high doses and promotes replication fork reversal (80). Fork reversal leads to the formation of a four stranded ‘chicken foot’ structure that can be targeted for resolution by structure specific nucleases such as MUS81 or GEN1 (85). As the influence of EXO1 was most apparent following treatment with low dose CPT, we analyzed survival following transient depletion of MUS81 that has been implicated in the processing of lesions generated by replication stress and influences cell death in number of contexts (86–88). Transient depletion of MUS81 enhanced the survival of wild type cells to a similar level as *Exo1* deficiency (Figure 6E and Supplementary Figure S7). Similarly, survival of MUS81 depleted *Nbs1^ΔB/ΔB^* cultures was rescued to a level similar to that of control siRNA treated *Nbs1^ΔB/ΔB^ Exo1^−/−^* cultures (Figure 6E). In contrast, MUS81 depletion had a relatively modest effect on the survival of *Nbs1^ΔB/ΔB^ Exo1^−/−^* cultures and caused almost no difference in the survival of *Exo1^−/−^* cells following low dose CPT treatment (Figure 6E and F). Collectively these results suggest that the processing of CPT lesions by EXO1 generates toxic intermediates and promotes cell death. This is in part appears to be due to the action of MUS81, as its depletion improves cellular survival to low dose CPT and this is more pronounced in cells that express EXO1 (Figure 6F).

## DISCUSSION

**Figure 7.**
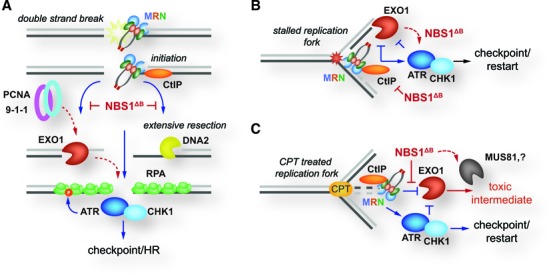
Proposed model of MRE11 complex and EXO1 interactions in replication and repair. (**A**) DSBs are recognized by the MRE11 complex (MRN) that initiates bidirectional resection through its nuclease activity. MRN promotes the exonuclease activity of both EXO1 and DNA2 that generate ssDNA that is bound by RPA. These RPA bound strands then promote the activation of ATR and CHK1 to promote checkpoint responses and homologous recombination. In cells expressing the NBS1^ΔB^ protein, the ability of MRN to promote EXO1 and DNA2 mediated resection is reduced and RPA phosphorylation is impaired. Resection becomes dependent on EXO1 and it may be recruited via PCNA or the 9-1-1 complex to promote ATR/CHK1 activation and suppress more severe checkpoint signaling and repair defects. (**B**) Stalled replication forks are recognized by both MRN and EXO1 and excessive resection is suppressed by feedback from ATR and CHK1. In cells expressing the NBS1^ΔB^ protein, EXO1 activity is increased, due to reduced negative feedback from ATR/CHK1, and it is able to compensate for defective MRN, thus suppressing more severe replication defects. (**C**) On forks that have stalled or regressed due to CPT, the combined actions of MRN and ATR/CHK1 may suppress the generation of intermediates by EXO1 that could be rendered cytotoxic by the actions of additional nucleases, such as MUS81.

We have sought to define the influence of EXO1 on the DDR to DSBs both on its own, as well as in cells with compromised MRE11 complex function. Consistent with some previous work in EXO1 deficient or siRNA depleted cells, we observe a mild increase in chromosomal instability and a minor reduction in RPA phosphorylation, but despite this, signaling responses and sensitivity to break inducing agents, such as IR or cisplatin, are comparable to wild type in cells lacking EXO1 (55). The lack of strong DSB induced phenotypes in EXO1 deficient cells likely reflects the ability of the MRE11 complex to promote the recruitment of additional factors, including DNA2, BLM and WRN, as has been reported in other systems (Figure 7A) (23,30,33,34,36–39). We speculate that the diverse genetic backgrounds of the cancer cell lines used in previous studies, or differential off-target effects, may account for the widely variable responses observed following EXO1 depletion by siRNA (60–66).

In contrast to the results in EXO1 null cells, we observed a strong dependency on EXO1 for the activation of ATM/ATR signaling, G2/M checkpoint induction and SSA in the hypomorphic *Nbs1^ΔB/ΔB^* background. The precise influence of the hypomorphic *Nbs1^ΔB^* allele on DSB responses has not been fully elucidated but the p80 protein that is produced, lacks the FHA and BRCT domains and is unstable, leading to a reduction in the amount and proper subcellular localization of MRE11 complex (4,67). Our previous data, as well as additional data presented here, suggests that the *Nbs1^ΔB^* mutation does not strongly impair DSB end capture and ATM activation, but does compromise resection efficiency, as indicated by reduced S4/8-phosphorylation of RPA and lower SSA efficiency (Figures 3G and 4C) (19). This is consistent with the possibility that EXO1 is less dependent on the FHA and BRCT domains of NBS1 (deleted in the NBS1^ΔB^/p80 protein) than complementary resection factors. The affinity of EXO1 for DNA ends is promoted by the MRE11 complex (64,89), but its resection activity and rapid damage recruitment is also potentiated by PCNA and the structurally related 9-1-1 complex, potentially providing numerous alternative recruitment options (40,42,90). In addition, our data suggests that EXO1 acts rapidly in *Nbs1^ΔB/ΔB^* cells, promoting the full activation of ATM (Figure 3E and F). We believe that limited EXO1 activity on gaps or replication structures may promote full ATM activation by enhancing positive feedback signaling following initial break detection, while more extensive resection is needed to promote the accumulation of ssDNA and RPA recruitment needed for optimal ATR-CHK1 signaling. This could potentially be due to enhanced RAD17 recruitment, as RAD17 has been shown to promote ATM activation via positive feedback at an early step, and would be expected to be reduced in the *Nbs1^ΔB/ΔB^* cells as interactions with the N-terminal domains, deleted in NBS1^ΔB^, have been implicated in its interaction with ATM (91).

Yeast that are doubly deficient for Mre11 complex members and Exo1 have a severe synthetic growth defect and *Exo1* overexpression suppresses the sensitivity of *mre11* null mutants to MMS, suggesting that MRE11 complex functions that are essential for cellular viability and damage repair can be carried out by *Exo1* (57,92). The same severe genetic interactions are not observed between the MRE11 complex and other key MMR proteins such as MSH2 and MSH6, suggesting that the genetic interaction is likely to be independent of EXO1's role in MMR (59). While *Nbs1^ΔB/ΔB^* mice have a mild developmental phenotype, the severe phenotypes of *Nbs1^ΔB/ΔB^ Exo1^−/−^* embryos and cell cultures, including growth defects, chromosomal instability and premature senescence, suggest that a similar relationship exists in mammals. As the MRE11 complex facilitates normal DNA synthesis and *Nbs1^ΔB/ΔB^ Exo1^−/−^* cultures exhibit severe growth defects, chromosomal instability and reduced replication fork velocity, it is likely that EXO1 is required to process aberrant replication structures generated during DNA replication in the *Nbs1^ΔB/ΔB^* background (Figure 7B). EXO1 has been shown to be negatively regulated both by interactions with CtIP and by ATR checkpoint responses and its activity may therefore be increased in the checkpoint defective *Nbs1^ΔB/ΔB^* background (64,93). This could reflect a role for EXO1 in MRE11 complex dependent DSB resection pathways, but it is also likely to extend to the processing of more complex replication structures that could arise in the context of physiological stress, such as reactive oxygen, during organismal development. This would be consistent with the observation that DSB sensitivity is only mildly enhanced by loss of EXO1 in *Nbs1^ΔB/ΔB^*, while the sensitivity to crosslinking agents, that typically generate more complex lesions in S-phase cells, is much higher in double mutants (Figure 5A and B).

Given the requirement of EXO1 for chromosome stability, DNA replication and break sensitivity in *Nbs1^ΔB/ΔB^* cultures, the fact that EXO1 deficiency promotes enhanced survival of these cells after treatment with low dose camptothecin was surprising. In contrast to what has been observed in human cancer cells, we are not able to detect global replication fork slowing or arrest at the doses used in these experiments (80). Thus directly monitoring fork stability and restart was not technically possible. By extrapolation from experiments with HU treatment, we believe that EXO1's negative influence on fork restart is consistent with a role in processing stalled forks and that this processing could lead to the generation of toxic intermediates directly or indirectly through additional nucleases such as MUS81 (Figure 7C). It is also noteworthy that we did not see fork restart defects in *Nbs1^ΔB/ΔB^* cultures, indicating that this mutation does not impair functions of the MRE11 complex required for restart, at least following HU treatment. We propose that EXO1 activity acts on replication fork structures induced by CPT, with regressed forks being a likely candidate. In normal cells, checkpoint arrest allows the repair of these lesions while the intra-S and G2/M defects of *Nbs1^ΔB/ΔB^* cells allow aberrant processing and their degradation or progression into mitosis. This may involve a number of nucleases that would normally be restrained by CDK inhibition but appears to be highly dependent on EXO1, as *Nbs1^ΔB/ΔB^ Exo1^−/−^* cultures showed a level of sensitivity near that of wild type.

The structure specific nuclease MUS81 has been shown to influence DNA damage sensitivity in checkpoint defective backgrounds and its depletion improved the sensitivity of all of the cell lines tested. However, despite similar levels of MUS81 knockdown, the effects on survival were more apparent in EXO1 proficient cells, indicating that EXO1 promotes the generation of at least some MUS81 substrates that result in the high toxicity of *Nbs1^ΔB/ΔB^* cells following low dose CPT treatment (Figures 5 and 6). The fact that the influence of EXO1 was much less apparent on the survival of *Nbs1^ΔB/ΔB^* cells at high dose CPT supports the likelihood that this is mainly independent of canonical DSB resection.

Collectively, our results demonstrate a robust genetic and functional relationship between EXO1 and the MRE11 complex in mammals and reveal EXO1 as an important modulator of S-phase progression and DNA damage sensitivity in an MRE11 complex hypomorphic background that shows otherwise relatively mild phenotypes in the absence of exogenous damage. As hypomorphic mutations of MRE11 complex members underlie several severe chromosomal instability disorders, this suggests that EXO1 may play a key role in suppressing the severity of the pathological outcomes associated with NBS and ATLD mutations and could genetically interact with less severe alleles of MRE11 complex members in multigenic hereditary disorders. In addition, as both EXO1 and members of the MRE11 complex are mutated in human malignancies, their relative status may be an important consideration regarding the efficacy of chemotherapeutic protocols involving DNA damaging agents.

## Supplementary Material

SUPPLEMENTARY DATA

## References

[1] Jackson S.P., Bartek J. (2009). The DNA-damage response in human biology and disease. Nature.

[2] Nussenzweig A., Nussenzweig M.C. (2010). Origin of chromosomal translocations in lymphoid cancer. Cell.

[3] Halazonetis T.D., Gorgoulis V.G., Bartek J. (2008). An oncogene-induced DNA damage model for cancer development. Science.

[4] Stracker T.H., Petrini J.H. (2011). The MRE11 complex: starting from the ends. Nat. Rev. Mol. Cell Biol..

[5] Stracker T.H., Roig I., Knobel P.A., Marjanovic M. (2013). The ATM signaling network in development and disease. Front. Genet..

[6] Paull T.T., Deshpande R.A. (2014). The Mre11/Rad50/Nbs1 complex: recent insights into catalytic activities and ATP-driven conformational changes. Exp. Cell Res..

[7] Rein K., Stracker T.H. (2014). The MRE11 complex: An important source of stress relief. Exp. Cell Res..

[8] Cannavo E., Cejka P. (2014). Sae2 promotes dsDNA endonuclease activity within Mre11-Rad50-Xrs2 to resect DNA breaks. Nature.

[9] Lengsfeld B.M., Rattray A.J., Bhaskara V., Ghirlando R., Paull T.T. (2007). Sae2 is an endonuclease that processes hairpin DNA cooperatively with the Mre11/Rad50/Xrs2 complex. Mol. Cell.

[10] Makharashvili N., Tubbs A.T., Yang S.H., Wang H., Barton O., Zhou Y., Deshpande R.A., Lee J.H., Lobrich M., Sleckman B.P. (2014). Catalytic and noncatalytic roles of the CtIP endonuclease in double-strand break end resection. Mol. Cell.

[11] Sartori A.A., Lukas C., Coates J., Mistrik M., Fu S., Bartek J., Baer R., Lukas J., Jackson S.P. (2007). Human CtIP promotes DNA end resection. Nature.

[12] Wang H., Li Y., Truong L.N., Shi L.Z., Hwang P.Y., He J., Do J., Cho M.J., Li H., Negrete A. (2014). CtIP maintains stability at common fragile sites and inverted repeats by end resection-independent endonuclease activity. Mol. Cell.

[13] Deshpande R.A., Williams G.J., Limbo O., Williams R.S., Kuhnlein J., Lee J.H., Classen S., Guenther G., Russell P., Tainer J.A. (2014). ATP-driven Rad50 conformations regulate DNA tethering, end resection, and ATM checkpoint signaling. EMBO J..

[14] Matsuoka S., Ballif B.A., Smogorzewska A., McDonald E.R. 3rd, Hurov K.E., Luo J., Bakalarski C.E., Zhao Z., Solimini N., Lerenthal Y. (2007). ATM and ATR substrate analysis reveals extensive protein networks responsive to DNA damage. Science.

[15] Mu J.J., Wang Y., Luo H., Leng M., Zhang J., Yang T., Besusso D., Jung S.Y., Qin J. (2007). A proteomic analysis of ataxia telangiectasia-mutated (ATM)/ATM-Rad3-related (ATR) substrates identifies the ubiquitin-proteasome system as a regulator for DNA damage checkpoints. J. Biol. Chem..

[16] Bensimon A., Schmidt A., Ziv Y., Elkon R., Wang S.Y., Chen D.J., Aebersold R., Shiloh Y. (2010). ATM-dependent and -independent dynamics of the nuclear phosphoproteome after DNA damage. Sci. Signal..

[17] Carson C.T., Schwartz R.A., Stracker T.H., Lilley C.E., Lee D.V., Weitzman M.D. (2003). The Mre11 complex is required for ATM activation and the G2/M checkpoint. EMBO J..

[18] Uziel T., Lerenthal Y., Moyal L., Andegeko Y., Mittelman L., Shiloh Y. (2003). Requirement of the MRN complex for ATM activation by DNA damage. EMBO J..

[19] Stracker T.H., Couto S.S., Cordon-Cardo C., Matos T., Petrini J.H. (2008). Chk2 suppresses the oncogenic potential of DNA replication-associated DNA damage. Mol. Cell.

[20] Stracker T.H., Morales M., Couto S.S., Hussein H., Petrini J.H. (2007). The carboxy terminus of NBS1 is required for induction of apoptosis by the MRE11 complex. Nature.

[21] Matsuoka S., Huang M., Elledge S.J. (1998). Linkage of ATM to cell cycle regulation by the Chk2 protein kinase. Science.

[22] Buscemi G., Savio C., Zannini L., Micciche F., Masnada D., Nakanishi M., Tauchi H., Komatsu K., Mizutani S., Khanna K. (2001). Chk2 activation dependence on Nbs1 after DNA damage. Mol. Cell. Biol..

[23] Shiotani B., Zou L. (2009). Single-stranded DNA orchestrates an ATM-to-ATR switch at DNA breaks. Mol. Cell.

[24] Duursma A.M., Driscoll R., Elias J.E., Cimprich K.A. (2013). A role for the MRN complex in ATR activation via TOPBP1 recruitment. Mol. Cell.

[25] Lee J., Dunphy W.G. (2013). The Mre11-Rad50-Nbs1 (MRN) complex has a specific role in the activation of Chk1 in response to stalled replication forks. Mol. Biol. Cell.

[26] Jazayeri A., Falck J., Lukas C., Bartek J., Smith G.C., Lukas J., Jackson S.P. (2006). ATM- and cell cycle-dependent regulation of ATR in response to DNA double-strand breaks. Nat. Cell Biol..

[27] Cimprich K.A., Cortez D. (2008). ATR: an essential regulator of genome integrity. Nat. Rev. Mol. Cell Biol..

[28] Zeman M.K., Cimprich K.A. (2014). Causes and consequences of replication stress. Nat. Cell Biol..

[29] Chapman J.R., Taylor M.R., Boulton S.J. (2012). Playing the end game: DNA double-strand break repair pathway choice. Mol. Cell.

[30] Shibata A., Moiani D., Arvai A.S., Perry J., Harding S.M., Genois M.M., Maity R., van Rossum-Fikkert S., Kertokalio A., Romoli F. (2014). DNA double-strand break repair pathway choice is directed by distinct MRE11 nuclease activities. Mol. Cell.

[31] Neale M.J., Pan J., Keeney S. (2005). Endonucleolytic processing of covalent protein-linked DNA double-strand breaks. Nature.

[32] Limbo O., Chahwan C., Yamada Y., de Bruin R.A., Wittenberg C., Russell P. (2007). Ctp1 is a cell-cycle-regulated protein that functions with Mre11 complex to control double-strand break repair by homologous recombination. Mol. Cell.

[33] Mimitou E.P., Symington L.S. (2008). Sae2, Exo1 and Sgs1 collaborate in DNA double-strand break processing. Nature.

[34] Zhu Z., Chung W.H., Shim E.Y., Lee S.E., Ira G. (2008). Sgs1 helicase and two nucleases Dna2 and Exo1 resect DNA double-strand break ends. Cell.

[35] Garcia V., Phelps S.E., Gray S., Neale M.J. (2011). Bidirectional resection of DNA double-strand breaks by Mre11 and Exo1. Nature.

[36] Sturzenegger A., Burdova K., Kanagaraj R., Levikova M., Pinto C., Cejka P., Janscak P. (2014). DNA2 cooperates with the WRN and BLM RecQ helicases to mediate long-range DNA end resection in human cells. J. Biol. Chem..

[37] Daley J.M., Chiba T., Xue X., Niu H., Sung P. (2014). Multifaceted role of the Topo IIIalpha-RMI1-RMI2 complex and DNA2 in the BLM-dependent pathway of DNA break end resection. Nucleic Acids Res..

[38] Nimonkar A.V., Genschel J., Kinoshita E., Polaczek P., Campbell J.L., Wyman C., Modrich P., Kowalczykowski S.C. (2011). BLM-DNA2-RPA-MRN and EXO1-BLM-RPA-MRN constitute two DNA end resection machineries for human DNA break repair. Genes Dev..

[39] Yang S.H., Zhou R., Campbell J., Chen J., Ha T., Paull T.T. (2012). The SOSS1 single-stranded DNA binding complex promotes DNA end resection in concert with Exo1. EMBO J..

[40] Chen X., Paudyal S.C., Chin R.I., You Z. (2013). PCNA promotes processive DNA end resection by Exo1. Nucleic Acids Res.

[41] Nielsen F.C., Jager A.C., Lutzen A., Bundgaard J.R., Rasmussen L.J. (2004). Characterization of human exonuclease 1 in complex with mismatch repair proteins, subcellular localization and association with PCNA. Oncogene.

[42] Liberti S.E., Andersen S.D., Wang J., May A., Miron S., Perderiset M., Keijzers G., Nielsen F.C., Charbonnier J.B., Bohr V.A. (2011). Bi-directional routing of DNA mismatch repair protein human exonuclease 1 to replication foci and DNA double strand breaks. DNA Repair.

[43] Mirzoeva O.K., Petrini J.H. (2003). DNA replication-dependent nuclear dynamics of the Mre11 complex. Mol. Cancer Res..

[44] Ying S., Hamdy F.C., Helleday T. (2012). Mre11-dependent degradation of stalled DNA replication forks is prevented by BRCA2 and PARP1. Cancer Res..

[45] Schlacher K., Christ N., Siaud N., Egashira A., Wu H., Jasin M. (2011). Double-strand break repair-independent role for BRCA2 in blocking stalled replication fork degradation by MRE11. Cell.

[46] Trenz K., Smith E., Smith S., Costanzo V. (2006). ATM and ATR promote Mre11 dependent restart of collapsed replication forks and prevent accumulation of DNA breaks. EMBO J..

[47] Hashimoto Y., Ray Chaudhuri A., Lopes M., Costanzo V. (2010). Rad51 protects nascent DNA from Mre11-dependent degradation and promotes continuous DNA synthesis. Nat. Struct. Mol. Biol..

[48] Bressan D.A., Baxter B.K., Petrini J.H. (1999). The Mre11-Rad50-Xrs2 protein complex facilitates homologous recombination-based double-strand break repair in Saccharomyces cerevisiae. Mol. Cell. Biol..

[49] Costanzo V., Robertson K., Bibikova M., Kim E., Grieco D., Gottesman M., Carroll D., Gautier J. (2001). Mre11 protein complex prevents double-strand break accumulation during chromosomal DNA replication. Mol. Cell.

[50] Cotta-Ramusino C., Fachinetti D., Lucca C., Doksani Y., Lopes M., Sogo J., Foiani M. (2005). Exo1 processes stalled replication forks and counteracts fork reversal in checkpoint-defective cells. Mol. Cell.

[51] Munoz-Galvan S., Lopez-Saavedra A., Jackson S.P., Huertas P., Cortes-Ledesma F., Aguilera A. (2013). Competing roles of DNA end resection and non-homologous end joining functions in the repair of replication-born double-strand breaks by sister-chromatid recombination. Nucleic Acids Res..

[52] Bruhn C., Zhou Z.W., Ai H., Wang Z.Q. (2014). The essential function of the MRN complex in the resolution of endogenous replication intermediates. Cell Rep..

[53] Wei K., Clark A.B., Wong E., Kane M.F., Mazur D.J., Parris T., Kolas N.K., Russell R., Hou H. Jr, Kneitz B. (2003). Inactivation of Exonuclease 1 in mice results in DNA mismatch repair defects, increased cancer susceptibility, and male and female sterility. Genes Dev..

[54] Tran P.T., Erdeniz N., Symington L.S., Liskay R.M. (2004). EXO1-A multi-tasking eukaryotic nuclease. DNA Repair.

[55] Schaetzlein S., Chahwan R., Avdievich E., Roa S., Wei K., Eoff R.L., Sellers R.S., Clark A.B., Kunkel T.A., Scharff M.D. (2013). Mammalian Exo1 encodes both structural and catalytic functions that play distinct roles in essential biological processes. Proc. Natl. Acad. Sci. U.S.A..

[56] Bolderson E., Richard D.J., Edelmann W., Khanna K.K. (2009). Involvement of Exo1b in DNA damage-induced apoptosis. Nucleic Acids Res..

[57] Moreau S., Morgan E.A., Symington L.S. (2001). Overlapping functions of the Saccharomyces cerevisiae Mre11, Exo1 and Rad27 nucleases in DNA metabolism. Genetics.

[58] Nakada D., Hirano Y., Sugimoto K. (2004). Requirement of the Mre11 complex and exonuclease 1 for activation of the Mec1 signaling pathway. Mol. Cell. Biol..

[59] Collins S.R., Miller K.M., Maas N.L., Roguev A., Fillingham J., Chu C.S., Schuldiner M., Gebbia M., Recht J., Shales M. (2007). Functional dissection of protein complexes involved in yeast chromosome biology using a genetic interaction map. Nature.

[60] Gravel S., Chapman J.R., Magill C., Jackson S.P. (2008). DNA helicases Sgs1 and BLM promote DNA double-strand break resection. Genes Dev..

[61] Polo S.E., Blackford A.N., Chapman J.R., Baskcomb L., Gravel S., Rusch A., Thomas A., Blundred R., Smith P., Kzhyshkowska J. (2012). Regulation of DNA-end resection by hnRNPU-like proteins promotes DNA double-strand break signaling and repair. Mol. Cell.

[62] Kousholt A.N., Fugger K., Hoffmann S., Larsen B.D., Menzel T., Sartori A.A., Sorensen C.S. (2012). CtIP-dependent DNA resection is required for DNA damage checkpoint maintenance but not initiation. J. Cell Biol..

[63] Karanja K.K., Cox S.W., Duxin J.P., Stewart S.A., Campbell J.L. (2012). DNA2 and EXO1 in replication-coupled, homology-directed repair and in the interplay between HDR and the FA/BRCA network. Cell Cycle.

[64] Eid W., Steger M., El-Shemerly M., Ferretti L.P., Pena-Diaz J., Konig C., Valtorta E., Sartori A.A., Ferrari S. (2010). DNA end resection by CtIP and exonuclease 1 prevents genomic instability. EMBO Rep..

[65] Bolderson E., Tomimatsu N., Richard D.J., Boucher D., Kumar R., Pandita T.K., Burma S., Khanna K.K. (2010). Phosphorylation of Exo1 modulates homologous recombination repair of DNA double-strand breaks. Nucleic Acids Res..

[66] Tomimatsu N., Mukherjee B., Deland K., Kurimasa A., Bolderson E., Khanna K.K., Burma S. (2012). Exo1 plays a major role in DNA end resection in humans and influences double-strand break repair and damage signaling decisions. DNA Repair.

[67] Williams B.R., Mirzoeva O.K., Morgan W.F., Lin J., Dunnick W., Petrini J.H. (2002). A murine model of Nijmegen breakage syndrome. Curr. Biol..

[68] Theunissen J.W., Petrini J.H. (2006). Methods for studying the cellular response to DNA damage: influence of the Mre11 complex on chromosome metabolism. Methods Enzymol..

[69] Bennardo N., Gunn A., Cheng A., Hasty P., Stark J.M. (2009). Limiting the persistence of a chromosome break diminishes its mutagenic potential. PLoS Genet..

[70] Jackson D.A., Pombo A. (1998). Replicon clusters are stable units of chromosome structure: evidence that nuclear organization contributes to the efficient activation and propagation of S phase in human cells. J. Cell Biol..

[71] Stracker T.H., Williams B.R., Deriano L., Theunissen J.W., Adelman C.A., Roth D.B., Petrini J.H. (2009). Artemis and nonhomologous end joining-independent influence of DNA-dependent protein kinase catalytic subunit on chromosome stability. Mol. Cell Biol..

[72] Brugmans L., Verkaik N.S., Kunen M., van Drunen E., Williams B.R., Petrini J.H., Kanaar R., Essers J., van Gent D.C. (2009). NBS1 cooperates with homologous recombination to counteract chromosome breakage during replication. DNA Repair.

[73] Shull E.R., Lee Y., Nakane H., Stracker T.H., Zhao J., Russell H.R., Petrini J.H., McKinnon P.J. (2009). Differential DNA damage signaling accounts for distinct neural apoptotic responses in ATLD and NBS. Genes Dev..

[74] Liu Q., Guntuku S., Cui X.S., Matsuoka S., Cortez D., Tamai K., Luo G., Carattini-Rivera S., DeMayo F., Bradley A. (2000). Chk1 is an essential kinase that is regulated by Atr and required for the G(2)/M DNA damage checkpoint. Genes Dev..

[75] Tsvetkov L., Xu X., Li J., Stern D.F. (2003). Polo-like kinase 1 and Chk2 interact and co-localize to centrosomes and the midbody. J. Biol. Chem..

[76] Wang H., Shi L.Z., Wong C.C., Han X., Hwang P.Y., Truong L.N., Zhu Q., Shao Z., Chen D.J., Berns M.W. (2013). The interaction of CtIP and Nbs1 connects CDK and ATM to regulate HR-mediated double-strand break repair. PLoS Genet..

[77] Huang X., Traganos F., Darzynkiewicz Z. (2003). DNA damage induced by DNA topoisomerase I- and topoisomerase II-inhibitors detected by histone H2AX phosphorylation in relation to the cell cycle phase and apoptosis. Cell Cycle.

[78] Mehta A., Haber J.E. (2014). Sources of DNA double-strand breaks and models of recombinational DNA repair. Cold Spring Harb. Perspect. Biol..

[79] Bryant H.E., Petermann E., Schultz N., Jemth A.S., Loseva O., Issaeva N., Johansson F., Fernandez S., McGlynn P., Helleday T. (2009). PARP is activated at stalled forks to mediate Mre11-dependent replication restart and recombination. EMBO J..

[80] Ray Chaudhuri A., Hashimoto Y., Herrador R., Neelsen K.J., Fachinetti D., Bermejo R., Cocito A., Costanzo V., Lopes M. (2012). Topoisomerase I poisoning results in PARP-mediated replication fork reversal. Nat. Struct. Mol. Biol..

[81] Polato F., Callen E., Wong N., Faryabi R., Bunting S., Chen H.T., Kozak M., Kruhlak M.J., Reczek C.R., Lee W.H. (2014). CtIP-mediated resection is essential for viability and can operate independently of BRCA1. J. Exp. Med..

[82] Evers B., Helleday T., Jonkers J. (2010). Targeting homologous recombination repair defects in cancer. Trends Pharmacol. Sci..

[83] Cavallo F., Graziani G., Antinozzi C., Feldman D.R., Houldsworth J., Bosl G.J., Chaganti R.S., Moynahan M.E., Jasin M., Barchi M. (2012). Reduced proficiency in homologous recombination underlies the high sensitivity of embryonal carcinoma testicular germ cell tumors to Cisplatin and poly (adp-ribose) polymerase inhibition. PLoS One.

[84] Moskwa P., Buffa F.M., Pan Y., Panchakshari R., Gottipati P., Muschel R.J., Beech J., Kulshrestha R., Abdelmohsen K., Weinstock D.M. (2011). miR-182-mediated downregulation of BRCA1 impacts DNA repair and sensitivity to PARP inhibitors. Mol. Cell.

[85] Sarbajna S., West S.C. (2014). Holliday junction processing enzymes as guardians of genome stability. Trends Biochem Sci..

[86] Fugger K., Chu W.K., Haahr P., Kousholt A.N., Beck H., Payne M.J., Hanada K., Hickson I.D., Sorensen C.S. (2013). FBH1 co-operates with MUS81 in inducing DNA double-strand breaks and cell death following replication stress. Nat. Communi..

[87] Jeong Y.T., Rossi M., Cermak L., Saraf A., Florens L., Washburn M.P., Sung P., Schildkraut C.L., Pagano M. (2013). FBH1 promotes DNA double-strand breakage and apoptosis in response to DNA replication stress. J. Cell Biol..

[88] Regairaz M., Zhang Y.W., Fu H., Agama K.K., Tata N., Agrawal S., Aladjem M.I., Pommier Y. (2011). Mus81-mediated DNA cleavage resolves replication forks stalled by topoisomerase I-DNA complexes. J. Cell Biol..

[89] Cannavo E., Cejka P., Kowalczykowski S.C. (2013). Relationship of DNA degradation by Saccharomyces cerevisiae exonuclease 1 and its stimulation by RPA and Mre11-Rad50-Xrs2 to DNA end resection. Proc. Natl. Acad. Sci. U.S.A..

[90] Ngo G.H., Balakrishnan L., Dubarry M., Campbell J.L., Lydall D. (2014). The 9-1-1 checkpoint clamp stimulates DNA resection by Dna2-Sgs1 and Exo1. Nucleic Acids Res..

[91] Wang Q., Goldstein M., Alexander P., Wakeman T.P., Sun T., Feng J., Lou Z., Kastan M.B., Wang X.F. (2014). Rad17 recruits the MRE11-RAD50-NBS1 complex to regulate the cellular response to DNA double-strand breaks. EMBO J..

[92] Tsubouchi H., Ogawa H. (2000). Exo1 roles for repair of DNA double-strand breaks and meiotic crossing over in Saccharomyces cerevisiae. Mol. Biol. Cell.

[93] El-Shemerly M., Hess D., Pyakurel A.K., Moselhy S., Ferrari S. (2008). ATR-dependent pathways control hEXO1 stability in response to stalled forks. Nucleic Acids Res..

