# Supplementary material for: EXO1 is critical for embryogenesis and the DNA damage response in mice with a hypomorphic Nbs1 allele
Source: Nucleic Acids Res. 2015 Jul 8;43(15):7371–87. doi: 10.1093/nar/gkv691 (PMC4551929; doi:10.1093/nar/gkv691)
Supplement: SUPPLEMENTARY DATA [file supp_43_15_7371__index.html]

EXO1 is critical for embryogenesis and the DNA damage response in mice with a hypomorphic Nbs1 allele — EXO1 is critical for embryogenesis and the DNA damage response in mice with a hypomorphic Nbs1 allele — SUPPLEMENTARY DATA 

# EXO1 is critical for embryogenesis and the DNA damage response in mice with a hypomorphic *Nbs1* allele

## SUPPLEMENTARY DATA

- SUPPLEMENTARY DATA
