# Supplementary material for: EXO1 is critical for embryogenesis and the DNA damage response in mice with a hypomorphic Nbs1 allele
Source: Nucleic Acids Res. 2015 Jul 8;43(15):7371–87. doi: 10.1093/nar/gkv691 (PMC4551929; doi:10.1093/nar/gkv691)
Supplement: SUPPLEMENTARY DATA [file supp_gkv691_nar-00596-d-2015-File009.pdf]

Supplementary figures and legends (NAR-00596-D-2015)

EXO1 is critical for embryogenesis and the DNA damage response in mice with a hypomorphic *Nbs1* allele

Katrin Rein, Diana A. Yanez, Berta Terre, Lluis Palenzuela, Suvi Aivio, Kaichun Wei, Winfried Edelmann, Jeremy M. Stark and Travis H. Stracker

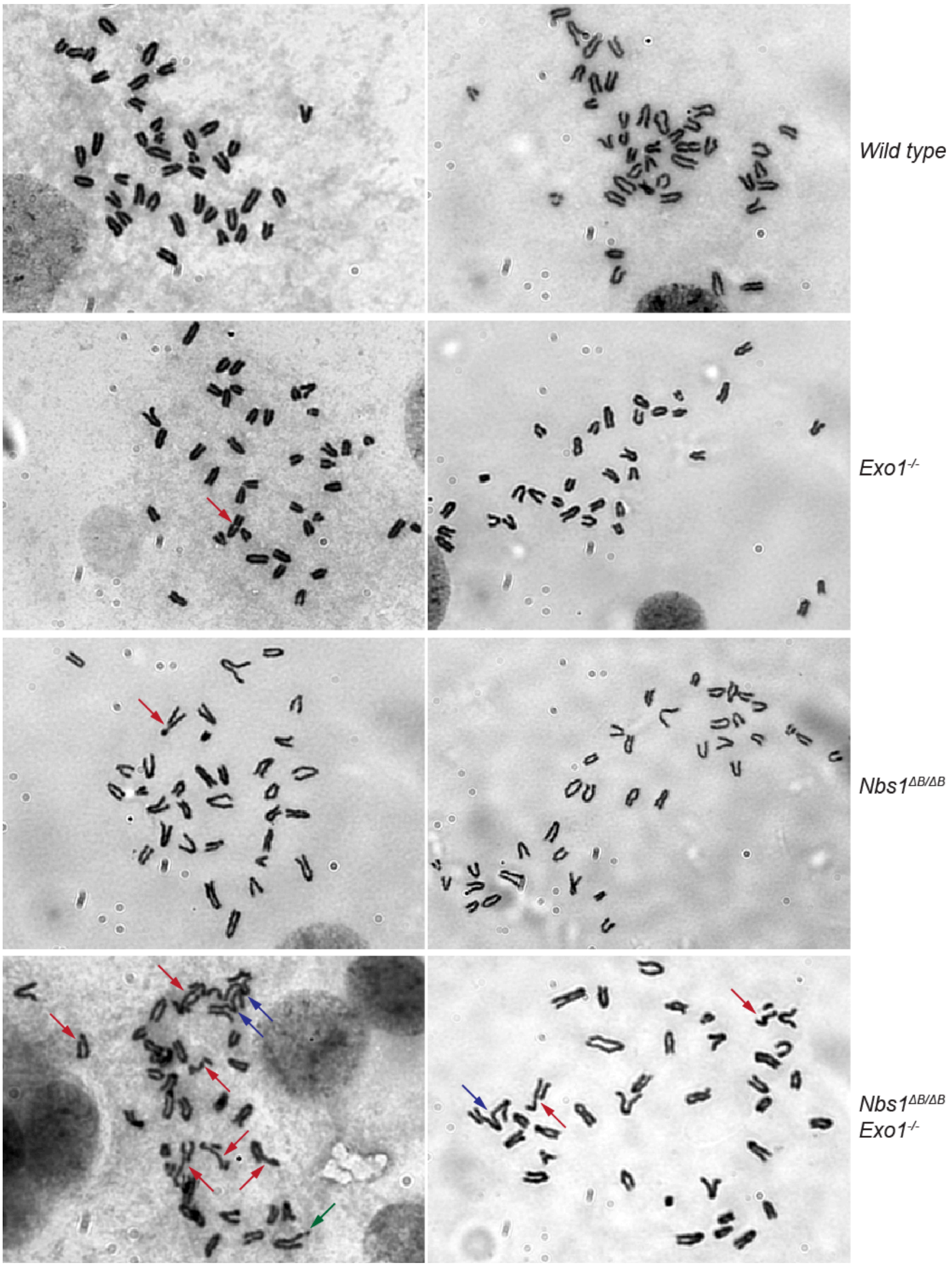

Figure S1: Examples of metaphase preparations. Metaphase chromosome preparations from the indicated genotypes are shown. Chromatid (cd) breaks (red arrows), fusions (blue arrows) and fragments (green arrows) are indicated.

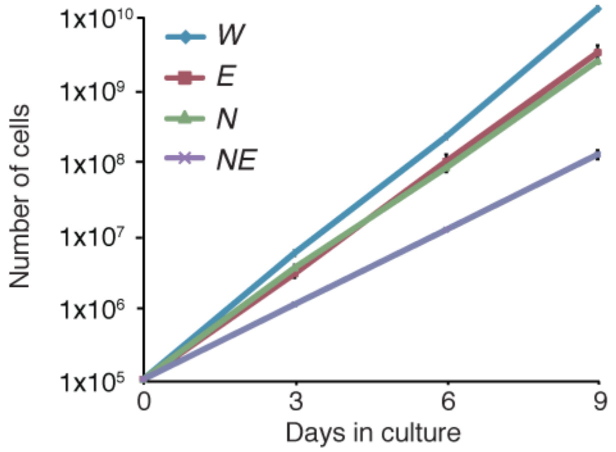

Figure S2: Cumulative cell growth of transformed cultures of the indicated genotype. Wild type = *W*, *Nbs1*$^{\Delta B/\Delta B}$, = *N*, *Exo1*$^{-/-}$ = *E*, *Nbs1*$^{\Delta B/\Delta B}$, and *Nbs1*$^{\Delta B/\Delta B}$ *Exo1*$^{-/-}$ = *NE*.

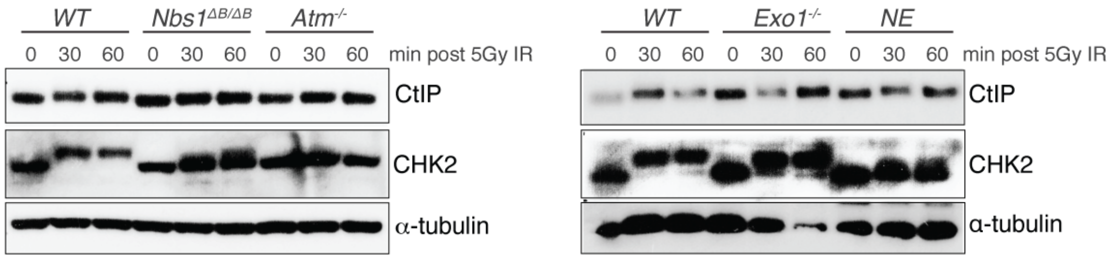

Figure S3: Western blotting of CtIP and CHK2 following 5 Gy IR treatment. Genotypes and times are indicated. Wild type = *WT and Nbs1*$^{\Delta B/\Delta B}$ *Exo*$^{-/-}$ = *NE.*

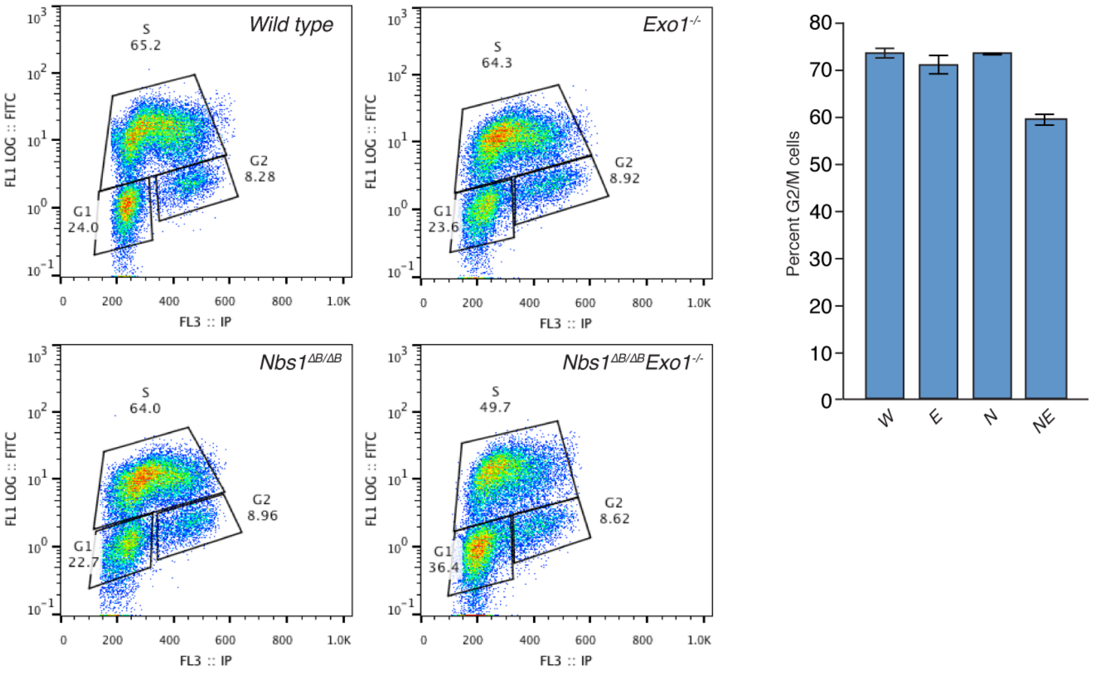

Figure S4: Analysis of cell cycle using BrdU and PI staining and flow cytometry in transformed MEF cultures of the indicated genotype. Plots of representative data are shown (left) and the mean and standard deviation for triplicates graphed (right). Genotypes are abbreviated as follows: wild type = *W*, *Nbs1*$^{\Delta B/\Delta B}$, = *N*, *Exo1*$^{-/-}$ = *E*, *Nbs1*$^{\Delta B/\Delta B}$, and *Nbs1*$^{\Delta B/\Delta B}$ *Exo*$^{-/-}$ = *NE.*

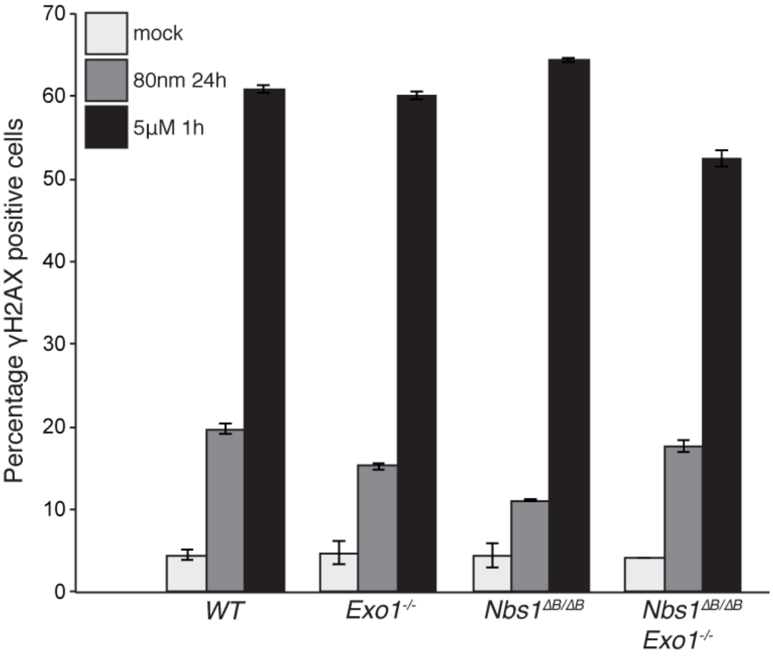

Figure S5: Assessment of the percentage of gH2AX positive cells by flow cytometry following the treatment of transformed cell cultures of the indicated genotype.

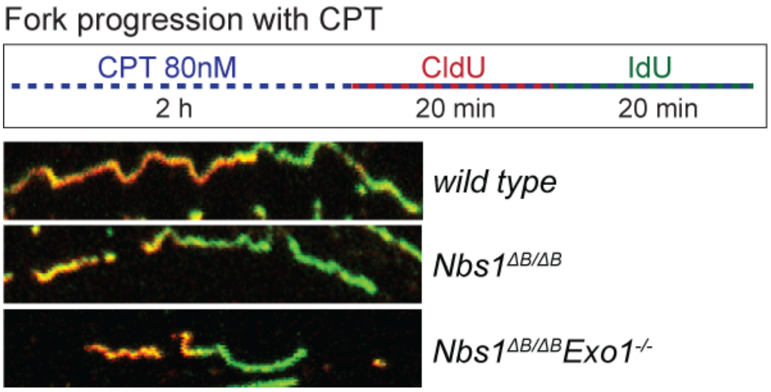

Figure S6: Examples of chromatin fiber spreads after CPT treatment. Forks from the fiber spreads from the indicated genotype following treatment with 80 nM CPT for 2 hours.

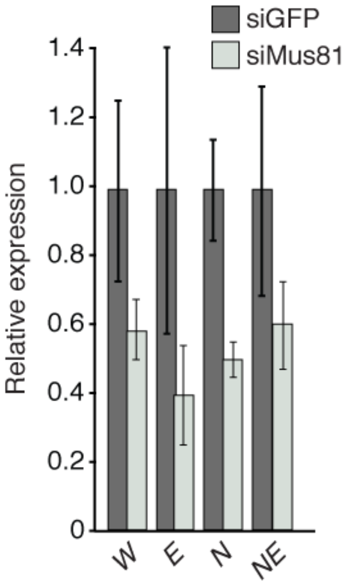

Figure S7: Real time PCR analysis of *Mus81* mRNA levels in cells of the indicated genotype transfected with siRNA against GFP or *Mus81*. Total RNA was extracted with lysis in Trizol and chloroform extraction. cDNA was prepared using the High Capacity cDNA Reverse Transcription Kit (ABI) using 2µg of RNA. mRNA levels of *Mus81* were determined by SYBR Green quantitative real-time PCR with GAPDH as an endogenous control with the following primers: MUS81-F: 5' TCCCTTCTTTCCAGATGGTG 3', MUS81-R: 5' ACTCCAGCACTTCGGAGACA 3', GAPDH-F: 5' GCACAGTCAAGGCCGAGAAT 3' and GAPDH-R: 5' GCCTTCTCCATGGTGGTGAA 3'. Genotypes are abbreviated as follows: wild type = *W*, *Nbs1*$^{\Delta B/\Delta B}$, = *N*, *Exo1*$^{-/-}$ = *E*, *Nbs1*$^{\Delta B/\Delta B}$, and *Nbs1*$^{\Delta B/\Delta B}$ *Exo*$^{-/-}$ = *NE*.
